# Tofacitinib as a maintenance therapy in patients with ulcerative colitis stratified by OCTAVE Sustain baseline Mayo endoscopic subscore

**DOI:** 10.1186/s12876-022-02508-2

**Published:** 2023-02-08

**Authors:** Scott D. Lee, Jessica R. Allegretti, Flavio Steinwurz, Susan B. Connelly, Nervin Lawendy, Jerome Paulissen, Krisztina B. Gecse

**Affiliations:** 1 Digestive Health Center, University of Washington Medical Center, Seattle, WA USA; 2 Division of Gastroenterology, Hepatology and Endoscopy, Brigham and Women’s Hospital, Boston, MA USA; 3 Unit of Inflammatory Bowel Disease, Hospital Israelita Albert Einstein, São Paulo, Brazil; 4 Pfizer Inc, Collegeville, USA; 5 Pfizer Inc, New York, NY USA; 6 Amsterdam UMC, Amsterdam, Netherlands

**Keywords:** Efficacy, Tofacitinib, Ulcerative colitis

## Abstract

**Background:**

Tofacitinib is an oral small molecule Janus kinase inhibitor for the treatment of ulcerative colitis. We evaluated tofacitinib efficacy and safety in the 52-week maintenance study, OCTAVE Sustain, by baseline Mayo endoscopic subscore (MES) following 8-week induction.

**Methods:**

The proportion of patients achieving efficacy endpoints at Week 24 or 52 of OCTAVE Sustain was evaluated by baseline MES following 8-week induction. Using logistic regression, the difference in treatment effect (tofacitinib vs. placebo) between baseline MES (0 vs. 1) for each endpoint was assessed. Adverse events were evaluated.

**Results:**

At Week 52 of OCTAVE Sustain, a numerically higher proportion of tofacitinib-treated patients achieved remission with OCTAVE Sustain baseline MES of 0 versus 1 (61.9% vs. 36.5% for tofacitinib 5 mg twice daily [BID] and 75.0% vs. 54.2% for tofacitinib 10 mg BID). Similar trends were observed for endoscopic remission and endoscopic improvement. Logistic regression analyses showed a larger treatment effect at Week 52 in patients with baseline MES of 0 versus 1 for clinical response (*p* = 0.0306) in the tofacitinib 5 mg BID group (other endpoints all *p* > 0.05); differences were not significant for any endpoint in the 10 mg BID group (all *p* > 0.05). Infection adverse events were less frequent among patients with baseline MES 0 versus 1.

**Conclusions:**

MES may be important in predicting long-term efficacy outcomes for tofacitinib maintenance treatment. Aiming for endoscopic remission during induction with tofacitinib 10 mg BID may allow successful maintenance with tofacitinib 5 mg BID. Safety was consistent with the known tofacitinib safety profile.

*Trial registration* NCT01458574.

## Background

Ulcerative colitis (UC) is a chronic disease characterized by relapsing and remitting mucosal inflammation of the rectum and colon [1]. Primary therapeutic goals are the achievement of clinical remission (cessation of rectal bleeding and normalization of bowel habits) and endoscopic improvement (commonly defined as a Mayo endoscopic subscore [MES] of 0 or 1 from a baseline of 2 or 3) [1].

Accumulating evidence indicates that endoscopic improvement is associated with better outcomes, including improved long-term remission rates and a decreased risk of colectomy [2]. However, a general consensus on the optimal MES cut-off (0 or 1) has yet to be determined [3], although the STRIDE initiative recently recommended complete endoscopic healing, defined by a MES of 0, as a treatment goal [4].

Several studies have compared long-term outcomes in patients with UC with endoscopic remission (MES 0) and endoscopic improvement (MES of 0 or 1). Patients with a MES of 0 have been shown to have a reduced risk of disease recurrence [5–7] and better health-related quality of life [8] compared with patients with a MES of 1.

Recent advances in the medical management of UC have made endoscopic improvement a realistic goal [9], and endoscopic indices are increasingly being used as an important measure of therapeutic efficacy in both clinical trials and real-world clinical practice [3]. However, no endoscopic marker has yet been established as an effective prognostic indicator of long-term response to biologic drugs, including tumor necrosis factor inhibitors (TNFi), in patients with UC [10]. The identification of such prognostic markers would help to better select for patients with a high probability of being responders to a particular drug, while minimizing the risks and costs involved for those who will likely be non-responders.

Tofacitinib is an oral small molecule Janus kinase inhibitor for the treatment of UC. The efficacy and safety of tofacitinib in patients with UC were demonstrated in two phase 3 induction trials (OCTAVE Induction 1 and 2) and a phase 3 maintenance trial (OCTAVE Sustain) [11]. Current guidance recommends using the lowest effective dose for tofacitinib maintenance; therefore, identifying predictors of safe and effective dose reduction of tofacitinib following induction would be beneficial [12, 13]. In this post hoc analysis of data from the OCTAVE Sustain study, we evaluated the efficacy and safety of tofacitinib 5 and 10 mg twice daily (BID) maintenance therapy in patients categorized according to baseline MES following 8-week induction therapy and prior TNFi exposure.

## Materials and methods

### Patients and study design

Full details of the OCTAVE Induction 1 and 2 (ClinicalTrials.gov; NCT01465763 and NCT01458951) and OCTAVE Sustain (ClinicalTrials.gov; NCT01458574) study designs were reported previously [11]. In OCTAVE Induction 1 and 2, patients with moderately to severely active UC, despite previous conventional or TNFi therapy, were randomized to receive tofacitinib induction therapy (10 mg BID) or placebo for 8 weeks. Patients in the tofacitinib or placebo groups who had a clinical response in OCTAVE Induction 1 and 2 (defined as a decrease from induction study baseline total Mayo score of ≥ 3 points and ≥ 30%, plus a decrease in rectal bleeding subscore of ≥ 1 point or an absolute rectal bleeding subscore of 0 or 1) were re-randomized to tofacitinib maintenance therapy (5 or 10 mg BID) or placebo for 52 weeks in OCTAVE Sustain. Induction non-responders and patients with loss of response or treatment failure in OCTAVE Sustain were eligible to enroll in an open-label, long-term extension study.

This post hoc analysis included patients in the full analysis set (FAS), defined as patients who received tofacitinib or placebo in OCTAVE Induction 1 and 2 and who were re-randomized to receive tofacitinib or placebo in OCTAVE Sustain, and who had a MES of 0 or 1 (based on central reading of endoscopy) at OCTAVE Sustain baseline (Week 8 of OCTAVE Induction 1 and 2).

The trial protocol for OCTAVE Sustain has previously been published [11] and was registered on ClinicalTrials.gov (NCT01458574) on 25/10/2011. As previously reported, all studies were conducted in compliance with the Declaration of Helsinki and the International Conference on Harmonization Good Clinical Practice Guidelines. Study protocols were approved by the Institutional Review Boards and/or independent ethics committees at each of the investigational centers participating in the studies, or a central Institutional Review Board [11]. All the patients provided written informed consent.

### Efficacy endpoints

As previously reported, efficacy endpoints at Week 24 or 52 of OCTAVE Sustain (based on central reading of endoscopy) included: remission (defined as a total Mayo score of ≤ 2 with no individual subscore > 1 and a rectal bleeding subscore of 0); endoscopic remission (MES of 0); endoscopic improvement (MES of 0 or 1); clinical response (defined above) [11]. Loss of response was defined by an increase in partial Mayo score of ≥ 2 points from OCTAVE Sustain baseline for two consecutive visits (at least 2 weeks apart), with an increase in rectal bleeding subscore of ≥ 1 from OCTAVE Sustain baseline [14]. Treatment failure was defined as an increase from OCTAVE Sustain baseline total Mayo score of ≥ 3 points, plus an increase in rectal bleeding subscore of ≥ 1 point and an increase in MES of ≥ 1 point yielding an absolute MES of ≥ 2, after a minimum of 8 weeks in the study [15].

### Safety assessments

Safety assessments included: adverse events (AEs) of special interest (serious infections, opportunistic infections [OIs], and herpes zoster [HZ; non-serious and serious]); gastrointestinal (GI) perforations; malignancies (excluding non-melanoma skin cancer [NMSC]); NMSC; major adverse cardiovascular events (MACE); venous thromboembolism (VTE); infections (all); and deaths. OIs, GI perforations, malignancies (including NMSC), and MACE were reviewed by independent adjudication committees.

### Analysis sets

The efficacy analysis set included patients in the FAS with non-missing binary efficacy endpoint responses (observed data). The time-to-event analysis set included all patients in the FAS (observed data). The safety analysis set included patients who received at least one dose of study medication in OCTAVE Sustain.

### Statistical analyses

The proportion of patients who achieved binary efficacy endpoints at Week 24 or 52 of OCTAVE Sustain was evaluated descriptively according to treatment group (tofacitinib 5 or 10 mg BID or placebo), OCTAVE Sustain baseline MES (0 vs. 1), and prior TNFi therapy.

In addition, the difference in treatment effect between baseline OCTAVE Sustain MES subgroups (0 vs. 1) was assessed using a logistic regression model, which included prior TNFi exposure status, treatment allocation, baseline MES, and treatment by baseline MES interaction. These analyses tested the null hypothesis that the tofacitinib 5 or 10 mg BID versus placebo difference (treatment effect) was the same for patients with a baseline MES of 0 as it was for those with a MES of 1.

Cox proportional hazards regression was used to model the difference in treatment effect between baseline MES (0 vs. 1) for time to treatment failure and time to loss of response. The model included prior TNFi exposure status, treatment allocation, baseline MES, and treatment by baseline MES interaction. These analyses tested whether the difference in the risk of the event between tofacitinib 5 or 10 mg BID and placebo was the same for patients with a baseline MES of 0 as it was for those with a baseline MES of 1.

Incidence rates, defined as the number of unique patients with events per 100 patient-years of exposure, were evaluated for AEs of special interest, which were counted up to 28 days beyond the last dose of study treatment. Confidence intervals (CIs) were calculated according to the Exact Poisson method, adjusted for patient-years.

## Results

### Patients

A total of 255 patients were included in the efficacy analysis set (patients in the FAS with non-missing binary efficacy endpoint responses [observed data]; tofacitinib 5 mg BID, n = 95; tofacitinib 10 mg BID, n = 75; placebo, n = 85). Prior TNFi exposure across the treatment groups among these 255 patients was 37.9% (36/95), 38.7% (29/75), and 45.9% (39/85), respectively. The time-to-event analysis set included a total of 295 patients (all patients in the FAS; tofacitinib 5 mg BID, n = 105; tofacitinib 10 mg BID, n = 89; placebo, n = 101). The safety analysis set included 294 patients who received at least one dose of study medication in OCTAVE Sustain (tofacitinib 5 mg BID, n = 105; tofacitinib 10 mg BID, n = 88; placebo, n = 101). Detailed baseline demographics and clinical characteristics of patients in OCTAVE Sustain were reported previously [11].

Demographics and baseline characteristics among all patients with OCTAVE Sustain baseline MES of 0 or 1 were generally similar between treatment groups and subgroups stratified by baseline MES, except for the proportion of patients in remission, total Mayo score, and the proportion of patients receiving corticosteroids at baseline of OCTAVE Sustain (Table 1). Across all treatment groups, the proportion of patients in remission was higher in patients with a baseline MES of 0 (80.0–88.5%) than in patients with a baseline MES of 1 (48.0–56.6%). Total Mayo score was lower in patients with a baseline MES of 0 versus 1 (0.9–1.5 vs. 2.4–2.7, respectively).

**Table 1:** Baseline demographic and clinical characteristics by treatment group and baseline MES

Variable	Tofacitinib 5 mg BID		Tofacitinib 10 mg BID		Placebo	
	MES = 0 (N = 22)	MES = 1 (N = 83)	MES = 0 (N = 20)	MES = 1 (N = 69)	MES = 0 (N = 26)	MES = 1 (N = 75)
Age (years), mean (SD)	36.5 (11.6)	41.9 (13.3)	39.5 (13.9)	42.0 (15.9)	42.3 (13.6)	43.4 (14.0)
Gender (female), n (%)	12 (54.5)	44 (53.0)	12 (60.0)	30 (43.5)	8 (30.8)	32 (42.7)
Race, n (%)						
White	17 (77.3)	72 (86.7)	13 (65.0)	51 (73.9)	21 (80.8)	63 (84.0)
Asian	3 (13.6)	7 (8.4)	4 (20.0)	11 (15.9)	2 (7.7)	7 (9.3)
Weight (kg), mean (SD)	79.1 (21.3)	72.9 (18.7)	71.9 (17.0)	72.0 (13.4)	76.0 (13.8)	77.7 (15.4)
BMI (kg/m^2^), mean (SD)	27.0 (7.1)	24.9 (5.0)	25.8 (5.3)	24.6 (4.0)	25.4 (5.1)	26.2 (4.7)
Remission at baseline, n (%)^a^	18 (81.8)	47 (56.6)	16 (80.0)	39 (56.5)	23 (88.5)	36 (48.0)
Total Mayo score at baseline, mean (SD)^a^	1.5 (1.3)	2.4 (1.2)	1.1 (1.0)	2.4 (1.1)	0.9 (1.2)	2.7 (1.4)
Corticosteroid use at baseline, n (%)^a^	8 (36.4)	53 (63.9)	11 (55.0)	27 (39.1)	13 (50.0)	37 (49.3)
5-ASA use at baseline, n (%)^a^	18 (81.8)	66 (79.5)	16 (80.0)	53 (76.8)	18 (69.2)	60 (80.0)
Prior TNFi exposure, n (%)^b^	7 (31.8)	33 (39.8)	6 (30.0)	29 (42.0)	8 (30.8)	38 (50.7)
Prior TNFi failure, n (%)^b^	4 (18.2)	30 (36.1)	5 (25.0)	27 (39.1)	8 (30.8)	36 (48.0)
Prior immunosuppressant failure, n (%)^b^	15 (68.2)	60 (72.3)	11 (55.0)	51 (73.9)	15 (57.7)	42 (56.0)
Extent of disease, n (%)^b^						
Proctosigmoiditis	4 (18.2)	16 (19.5)	5 (25.0)	10 (14.5)	2 (7.7)	11 (14.7)
Left-sided colitis	5 (22.7)	31 (37.8)	9 (45.0)	22 (31.9)	12 (46.2)	23 (30.7)
Extensive colitis/pancolitis	13 (59.1)	35 (42.7)	6 (30.0)	37 (53.6)	12 (46.2)	40 (53.3)
Proctitis	0 (0.0)	0 (0.0)	0 (0.0)	0 (0.0)	0 (0.0)	1 (1.3)

MES, based on central read of endoscopy, was assessed at Week 8 of OCTAVE Induction 1 or 2 (baseline of OCTAVE Sustain)

*5-ASA* 5-aminosalicylates; *BID* Twice daily; *BMI* Body mass index; *MES* Mayo endoscopic subscore; *N* Number of evaluable patients in the treatment group and MES subgroup; *n* Number of patients within the given category; *SD* Standard deviation; *TNFi* Tumor necrosis factor inhibitor

^a^Data from baseline of OCTAVE Sustain

^b^Data from baseline of OCTAVE Induction 1 or 2

Across all treatment groups in OCTAVE Sustain, there were similar proportions of patients with a baseline MES of 0 (tofacitinib 5 mg BID, 21.0%; tofacitinib 10 mg BID, 22.5%; placebo, 25.7%).

### Efficacy endpoints at Week 24 or Week 52 of OCTAVE Sustain

**Fig. 1:**
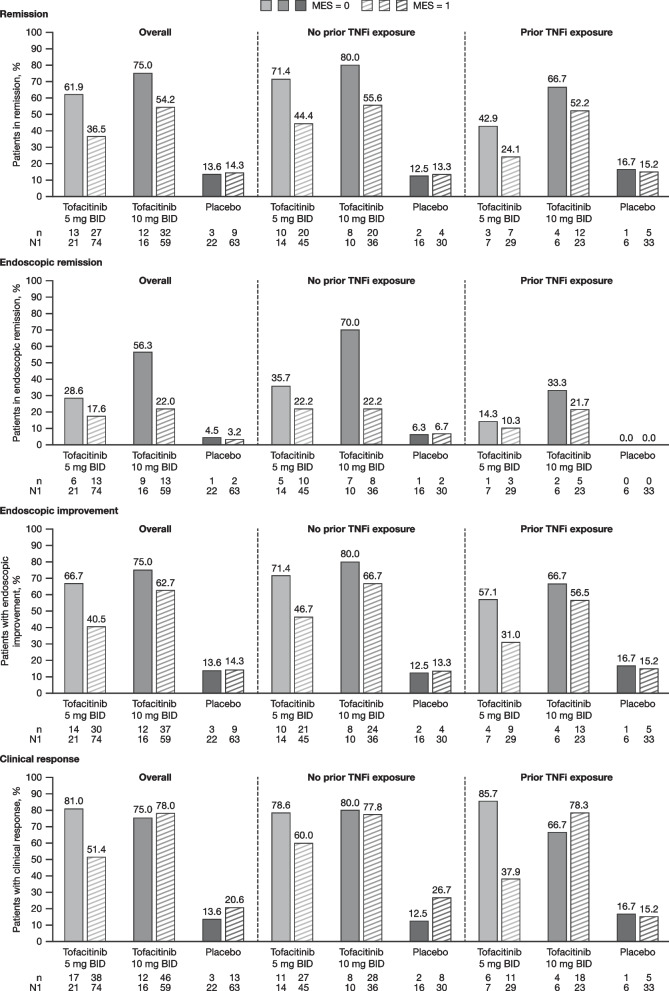
Proportion of patients achieving efficacy endpoints^a^ by baseline MES, overall and by prior TNFi exposure. MES, based on central read of endoscopy, was assessed at Week 8 of OCTAVE Induction 1 or 2 (baseline of OCTAVE Sustain). Only patients who had a MES of 0 or 1 at Week 8 of OCTAVE Induction 1 or 2 are included in this analysis. ^a^At Week 52 of OCTAVE Sustain (FAS, observed data). BID, twice daily; MES, Mayo endoscopic subscore; FAS, full analysis set; n, number of patients meeting the endpoint criteria; N1, number of patients in each subgroup with non-missing data; TNFi, tumor necrosis factor inhibitor

**Fig. 2:**
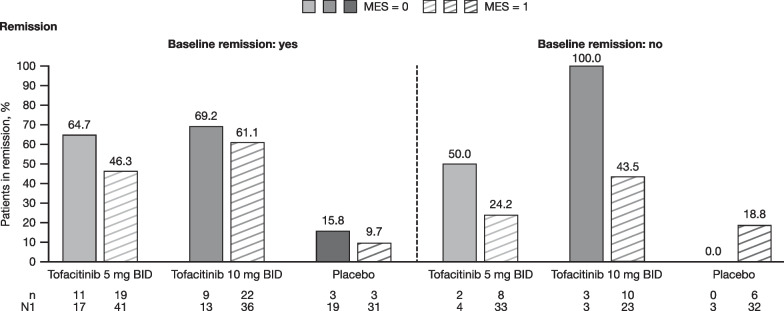
Proportion of patients in remission^a^ by baseline remission status, and baseline MES. MES, based on central read of endoscopy, was assessed at Week 8 of OCTAVE Induction 1 or 2 (baseline of OCTAVE Sustain). Only patients who had a MES of 0 or 1 at Week 8 of OCTAVE Induction 1 or 2 are included in this analysis. ^a^At Week 52 of OCTAVE Sustain (FAS, observed data). BID, twice daily; FAS, full analysis set; MES, Mayo endoscopic subscore; n, number of patients meeting the endpoint criteria; N1, number of patients in each treatment group with non-missing data

Overall, the proportion of patients who met efficacy endpoints was higher with tofacitinib than with placebo, regardless of tofacitinib dose or baseline MES. In general, the proportion of patients treated with tofacitinib who achieved efficacy endpoints at Week 52 was numerically higher for patients with a baseline MES of 0 than for those with a MES of 1. This was observed for remission, endoscopic remission, and endoscopic improvement, regardless of dose or prior TNFi exposure status (Fig. 1). For clinical response, the proportion was numerically higher for patients with a baseline MES of 0 versus 1 in the tofacitinib 5 mg BID group, but not in the tofacitinib 10 mg BID group. A numerically higher proportion of tofacitinib-treated patients achieved remission with a baseline MES of 0 versus 1 at Week 52 of OCTAVE Sustain (61.9% vs. 36.5% for tofacitinib 5 mg BID and 75.0% vs. 54.2% for tofacitinib 10 mg BID, respectively). However, at Week 24 this trend was only evident in the tofacitinib 5 mg BID group; the proportion of tofacitinib-treated patients who achieved remission at Week 24 with a baseline MES of 0 versus 1 was 76.2% versus 38.7% for tofacitinib 5 mg BID and 55.6% versus 58.5% for tofacitinib 10 mg BID, respectively. A similar trend of a higher proportion of tofacitinib-treated patients in remission at Week 52 with a baseline MES of 0 versus 1 was also observed irrespective of remission status at baseline of OCTAVE Sustain, although data were limited by small patient numbers in some groups (Fig. 2).

Logistic regression analyses of clinical response at Week 52 showed a significantly larger tofacitinib versus placebo treatment effect for patients with a baseline MES of 0 versus 1 in the tofacitinib 5 mg BID group (odds ratio [OR] [95% CI] 7.41 [1.21–45.52]; *p* = 0.0306) but not in the tofacitinib 10 mg BID group (OR 1.56 [0.24–10.33]; *p* = 0.6419). The difference in the tofacitinib 5 mg BID treatment effect between baseline MES of 0 and 1 was not evident for remission (OR 3.29 [0.58–18.70]; *p* = 0.1790), endoscopic remission (OR 1.51 [0.10–22.67]; *p* = 0.7655), or endoscopic improvement (OR 3.38 [0.59–19.40]; *p* = 0.1720). Similarly, the treatment effect of tofacitinib 10 mg BID was not significantly different between baseline MES 0 and 1 for any efficacy endpoint: remission, OR 3.00 (0.45–19.86); *p* = 0.2546; endoscopic remission, OR 3.94 (0.25–60.81); *p* = 0.3265; or endoscopic improvement, OR 2.08 (0.31–13.81); *p* = 0.4475.

**Fig. 3:**
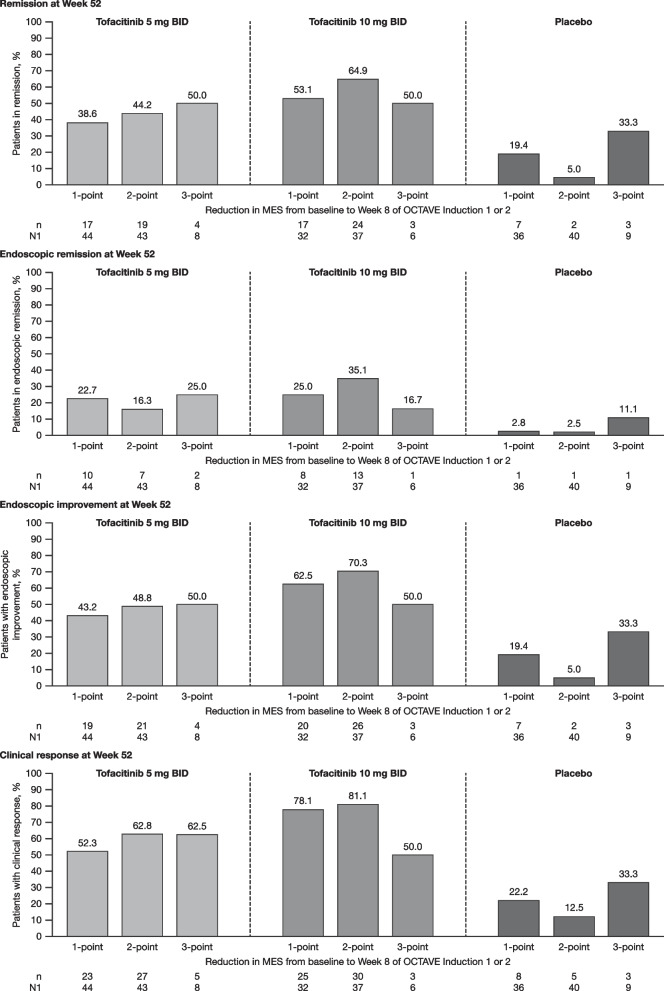
Proportion of patients achieving efficacy endpoints^a^ by reduction in MES^b^ during 8-week induction. MES, based on central read of endoscopy, was assessed at baseline and Week 8 of OCTAVE Induction 1 or 2. Only patients who had a MES of 0 or 1 at Week 8 of OCTAVE Induction 1 or 2 are included in this analysis. ^a^At Week 52 of OCTAVE Sustain (FAS, observed data). ^b^From baseline to Week 8 of OCTAVE Induction 1 or 2. BID, twice daily; MES, Mayo endoscopic subscore; FAS, full analysis set; n, number of patients meeting the endpoint criteria; N1, number of patients in each subgroup with non-missing data

We next examined the proportion of patients achieving efficacy endpoints at Week 52 of OCTAVE Sustain based on the extent of change in MES from baseline to Week 8 of OCTAVE Induction 1 or 2 (Fig. 3). The proportion of tofacitinib-treated patients achieving efficacy endpoints in OCTAVE Sustain was similar for patients with a 1-point or 2-point change in MES from baseline to Week 8 of OCTAVE Induction 1 or 2, although there was a general trend for the proportion of patients meeting each endpoint to be slightly higher in patients with a 2-point change versus those who had a 1-point change. Data for patients with a 3-point change in MES from baseline to Week 8 of OCTAVE Induction 1 or 2 were limited by small patient numbers.

**Fig. 4:**
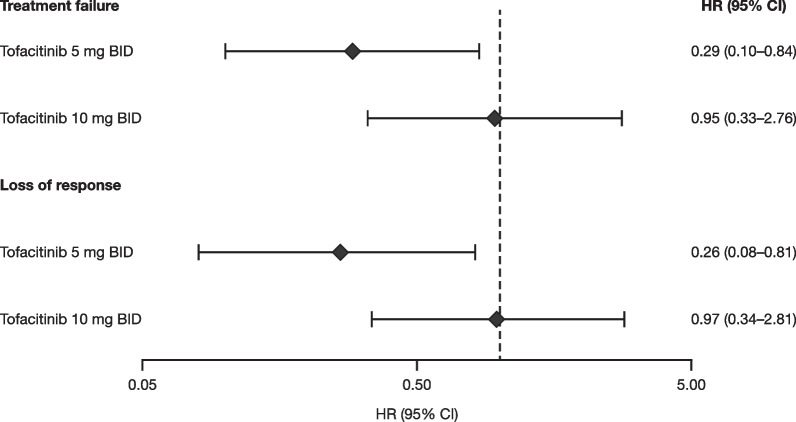
Cox proportional hazards regression^a^ modeling risk versus placebo according to baseline^b^ MES (0 vs. 1). Data represent the time-to-event analysis set (patients in the FAS with OCTAVE Sustain baseline MES of 0 or 1; observed data), which included a total of 295 patients (tofacitinib 5 mg BID, n = 105; tofacitinib 10 mg BID, n = 89; placebo, n = 101). ^a^Cox proportional hazards regression was used to model the difference in treatment effect (tofacitinib vs. placebo risk) between baseline MES (0 vs. 1) for time to treatment failure and time to loss of response. The model included prior TNFi exposure status, treatment allocation, baseline MES, and treatment by baseline MES interaction. ^b^Baseline of OCTAVE Sustain. BID, twice daily; CI, confidence interval; FAS, full analysis set; HR, hazard ratio, MES, Mayo endoscopic subscore; n, number of patients in each treatment group; TNFi, tumor necrosis factor inhibitor

For patients treated with tofacitinib 5 mg BID, Cox proportional hazards regression showed that the relative risk (tofacitinib vs. placebo) of treatment failure or loss of response was significantly lower for patients with a baseline MES 0 versus 1 (hazard ratio [95% CI] for MES 0 vs. 1: 0.29 [0.10–0.84]; *p* = 0.0231 for treatment failure and 0.26 [0.08–0.81]; *p* = 0.0209 for loss of response) (Fig. 4). In contrast, for patients treated with tofacitinib 10 mg BID, the risk versus placebo was not significantly different between baseline MES 0 versus 1 for treatment failure or loss of response (Fig. 4).

### Safety

Among patients receiving tofacitinib 5 mg BID in OCTAVE Sustain, a lower proportion of patients with a baseline MES of 0 versus 1 had infection AEs (all) (22.7% vs. 45.8%). This trend was weaker in the placebo group (19.2% vs. 29.3%) and among patients receiving tofacitinib 10 mg BID (35.0% vs. 39.7%). AEs of special interest are summarized in Table 2. The proportions of patients with OIs or HZ (non-serious and serious) were low across treatment and MES subgroups. No cases of serious infection, malignancy (including NMSC), MACE, GI perforation, or VTE (including deep vein thrombosis and/or pulmonary embolism) were reported in either tofacitinib treatment group. No deaths were reported in any treatment group.

**Table 2:** Summary of AEs of special interest by treatment group and baseline MES

	Tofacitinib 5 mg BID		Tofacitinib 10 mg BID		Placebo	
	MES = 0 (N = 22)	MES = 1 (N = 83)	MES = 0 (N = 20)	MES = 1 (N = 68)	MES = 0 (N = 26)	MES = 1 (N = 75)
Serious infections, n (%)	0 (0)	0 (0)	0 (0)	0 (0)	0 (0)	1 (1.3)
IR (95% CI)	0 (0–18.9)	0 (0–6.0)	0 (0–22.7)	0 (0–6.3)	0 (0–27.2)	2.4 (0.1–13.1)
Opportunistic infections, n (%)^a,b^	0 (0)	1 (1.2)	1 (5.0)	1 (1.5)	0 (0	0 (0)
IR (95% CI)	0 (0–18.9)	1.7 (0.0–9.2)	6.6 (0.2–36.5)	1.8 (0.0–9.7)	0 (0–27.2)	0 (0–8.7)
HZ (non-serious and serious), n (%)	0 (0)	2 (2.4)	2 (10.0)	2 (2.9)	0 (0)	0 (0)
IR (95% CI)	0 (0–18.9)	3.4 (0.4–12.1)	14.0 (1.7–50.6)	3.5 (0.4–12.7)	0 (0–27.2)	0 (0–8.7)
GI perforations, n (%)^a,c^	0 (0)	0 (0)	0 (0)	0 (0)	0 (0)	1 (1.3)
IR (95% CI)	0 (0–18.9)	0 (0–6.0)	0 (0–22.7)	0 (0–6.3)	0 (0–27.2)	2.4 (0.1–13.1)
Malignancies (excl. NMSC), n (%)^a^	0 (0)	0 (0)	0 (0)	0 (0)	0 (0)	1 (1.3)
IR (95% CI)	0 (0–18.9)	0 (0–6.0)	0 (0–22.7)	0 (0–6.3)	0 (0–27.2)	2.4 (0.1–13.1)
NMSC, n (%)^a^	0 (0)	0 (0)	0 (0)	0 (0)	0 (0)	0 (0)
IR (95% CI)	0 (0–18.9)	0 (0–6.0)	0 (0–22.7)	0 (0–6.3)	0 (0–27.2)	0 (0–8.7)
MACE, n (%)^a^	0 (0)	0 (0)	0 (0)	0 (0)	0 (0)	0 (0)
IR (95% CI)	0 (0–18.9)	0 (0–6.0)	0 (0–22.7)	0 (0–6.3)	0 (0–27.2)	0 (0–8.7)
VTE, n (%)	0 (0)	0 (0)	0 (0)	0 (0)	0 (0)	0 (0)
IR (95% CI)	0 (0–18.9)	0 (0–6.0)	0 (0–22.7)	0 (0–6.3)	0 (0–27.2)	0 (0–8.7)

MES, based on central read of endoscopy, was assessed at Week 8 of OCTAVE Induction 1 or 2 (baseline of OCTAVE Sustain). Only patients who had a MES of 0 or 1 at Week 8 of OCTAVE Induction 1 or 2 are included in this analysis. Data represent the safety analysis set (patients who received at least one dose of study medication in OCTAVE Sustain), which included 294 patients (tofacitinib 5 mg BID, N = 105; tofacitinib 10 mg BID, N = 88; placebo, N = 101)

*AE* Adverse event; *BID* Twice daily; *CI* Confidence interval; *GI* Gastrointestinal; *HZ* Herpes zoster; *IR* Incidence rate (number of patients with events per 100 patient-years of exposure); *MACE* Major adverse cardiovascular event; *MES* Mayo endoscopic subscore; *N* Number of patients in the specified category; *n* Number of patients with the specified safety event; *NMSC* Non-melanoma skin cancer; *VTE* Venous thromboembolism

^a^Adjudicated events

^b^Excludes tuberculosis and HZ with two adjacent dermatomes

^c^Excludes preferred terms of pilonidal cyst, perirectal abscess, rectal abscess, anal abscess, perineal abscess, and any preferred terms using the term fistula

## Discussion

In this post hoc analysis of data from the phase 3 OCTAVE Sustain study, we examined the efficacy and safety of tofacitinib maintenance therapy in patients categorized according to OCTAVE Sustain baseline MES to determine whether a MES of 0 (endoscopic remission) following 8-week induction therapy is associated with superior long-term outcomes at 52 weeks compared with a MES of 1 in patients with moderate-to-severe UC.

In general, a numerically higher proportion of tofacitinib-treated patients with a baseline MES of 0 at the start of maintenance therapy achieved efficacy endpoints at Week 52 compared to patients with a baseline MES of 1. This trend was observed regardless of prior TNFi exposure status or remission status at baseline of OCTAVE Sustain. When the proportion of patients achieving efficacy endpoints at Week 52 was stratified by change in MES from baseline to Week 8 of OCTAVE Induction 1 or 2, there was a slight trend for values to be numerically higher in patients who had a 2-point reduction in MES compared with those who had a 1-point reduction in MES. These results are broadly consistent with those of previous studies, which showed that patients with a MES of 0 have better outcomes compared with patients with a MES of 1, including a reduced risk of disease recurrence [5–7, 16], a higher rate of clinical remission [17], and better health-related quality of life [8].

In the tofacitinib 5 mg BID group, Week 52 results for clinical response showed a significant difference in the tofacitinib treatment effect for a baseline MES of 0 versus 1; however, this difference was not significant for remission, endoscopic remission, or endoscopic improvement in the 5 mg BID group or for any endpoint in the 10 mg BID group. This lack of statistical significance could at least in part be due to the small sample sizes, as a numerical trend was observed for most endpoints. In addition, for the tofacitinib 5 mg BID group, the difference in risk of treatment failure and loss of response versus placebo was significantly larger in patients with a baseline MES of 0 compared with those with a MES of 1. These differences were also not seen in the tofacitinib 10 mg BID group. These results suggest that remaining on the induction dose of tofacitinib 10 mg BID until endoscopic remission (MES of 0) is achieved may improve the probability of long-term efficacy with the 5 mg BID maintenance dose. This predictor of safe and effective dose reduction is particularly beneficial as current guidance recommends using the lowest effective dose for tofacitinib maintenance [12, 13].

Safety outcomes were consistent with the known safety profile of tofacitinib in UC, irrespective of baseline MES status. However, among patients receiving tofacitinib 5 mg BID, a lower proportion of patients with a baseline MES of 0 had infection AEs, compared with patients with a MES of 1. This trend was weaker but present in the tofacitinib 10 mg BID and placebo groups. This may suggest that patients with inflammatory burden, as suggested by a MES of 1, may be more susceptible to infection than those with a MES of 0 [18, 19]. However, this finding was based on a relatively small sample size and should be interpreted with caution.

Noted limitations of this analysis include the post hoc exploratory nature of the analyses and the small sample size, particularly of the TNFi exposure subgroups. Another limitation of these analyses is that the population of patients in the tofacitinib UC program may not be fully representative of the global UC population. It should also be noted that differences in baseline corticosteroid use between treatment groups and MES subgroups may have contributed to the observed trends for the tofacitinib 10 and 5 mg BID groups.

Additionally, inherent limitations of the MES include the lack of validation, the inability to distinguish superficial ulcers from deep ulcers, and the fact that the score only evaluates the most severely affected visualized segment, with no minimal insertion length. The MES is also limited by subjectivity and potential operator variability, although central reading was employed in this study to minimize any potential bias. While confirmation of remission status via histological assessment would further improve the reliability of these results, histological data were not collected in the OCTAVE clinical programme as these studies pre-dated the current FDA guidance on the inclusion of a histological assessment as an exploratory endpoint.

In conclusion, these analyses suggest that MES at the end of induction therapy may be an important factor in predicting long-term efficacy outcomes following maintenance treatment with tofacitinib. Aiming for endoscopic remission during induction with tofacitinib 10 mg BID may improve the probability of successful maintenance therapy with tofacitinib 5 mg BID.

## Data Availability

The datasets generated and analyzed during the current study are not publicly available in order to ensure the protection of patient privacy in compliance with the EU General Data Protection Regulation (GDPR) but are available from the corresponding author on reasonable request.

## Abbreviations

5-ASA: 5-aminosalicylates
AE: Adverse event
BID: Twice daily
BMI: Body mass index
CI: Confidence interval
FAS: Full analysis set
GI: Gastrointestinal
HR: Hazard ratio
HZ: Herpes zoster
IR: Incidence rate
MACE: Major adverse cardiovascular events
MES: Mayo endoscopic subscore
N1: Number of patients in each subgroup with non-missing data
NMSC: Non-melanoma skin cancer
OI: Opportunistic infection
OR: Odds ratio
SD: Standard deviation
TNFi: Tumor necrosis factor inhibitor
UC: Ulcerative colitis
VTE: Venous thromboembolism
